# Annals of Quackery—I

**Published:** 1910-06-11

**Authors:** 


### SPECIAL ARTICLES.

### ANNALS OF QUACKERY—I.

Many years ago—to be precise, in the year 1824—a strenuous campaign began to be waged by a public journal against quackery, and continued each week without abatement for eighteen months, at the end of which time it suddenly ceased, under circumstances which shall be related in due course. In so many respects were the methods of quacks in those days similar to those which their successors practise to-day that some account of this relentless campaign will be of peculiar interest at the present time.

### Jacobean Punishments.

In an early issue it is set forth as a matter of history that in the reign of James I. an Order in Council, founded on former laws, was issued for the apprehension of all quacks, in order to their being examined by the censors of the College of Physicians; on that occasion "several mountebanks, water tasters, ague charmers, and vendors of nostrums were fined, imprisoned, and banished." This wholesome severity, it may be supposed, checked the evil for a time; but in the reign of William III. it became again necessary to put the laws in force against "these base vermin and miscreants," in consequence of which many of them were examined, confessed their utter ignorance to such a degree as to be unable either to read or write; others, it was found, had been attempting to procure abortion in unfortunate single women; several of them were discovered to be "fortune tellers, matchmakers, frauders, pimps, and bawds." Some of these miscreants were set in the pillory, some put on horseback "with their faces to the horse's tail, with their noses and lips split, and their necks decorated with a collar of urinals, and afterwards whipped, imprisoned, branded, and banished." Whether these historical details were recalled as a warning does not appear, but it will no doubt interest the quacks even of the present day to know what sort of treatment was meted out to their forbears.

### A Classic Exposure.

To resume the story of this singular campaign, it may be stated at the outset that, like so many others, it was almost fruitless, although the journal itself by no means entertained this view; on the contrary, after twenty-eight weeks of existence it went so far as to say that when it began its career "the hydra was raging with unchecked rapacity, undermining the dearest possession of a simple and confiding portion of the public, devouring their life-blood; yet the monster, shorn of many of its heads and stabbed at its most vital part, instead of mounting high and insolent, now drags its wounded length along, and looks with the yellowness of despair upon its speedy and certain destruction. Another session of Parliament, it is hoped, will put an end to quackery." We who are living nearly ninety years later know that the hydra is still as "high and insolent" as ever, and that the writer was deceived. The paper was called the *Medical Adviser*, and although it appears to have enjoyed a large circulation during its brief life, it is rare that a copy is to be met with nowadays, and, for that reason, this the most striking and vigorous of all attacks against quackery has been overlooked. It goes without saying that threats of libel actions against the publishers were of frequent occurrence, and, in fact, in an early issue we find the following: "One of these quacks has sent us indirectly a threat of indictment for libel! . . . How can such fellows hope to have the indulgence of a verdict of honest men in their favour?" and a little later: "This wretched miscreant has threatened our publisher with an action!" In a later issue appears the statement that a club of quacks has been formed to attack the paper, and a fund subscribed. Notwithstanding these threats the *Medical Adviser* week by week continued to expose one quack after another, giving precise details of their methods and their antecedents; but before selecting specimens from these specific attacks, which are, by the way, most vivid, it will be of interest to quote from some of the expressions of opinion on quackery in general. For instance, how applicable to the present day is the following, which appeared in the issue of March 20, 1824: "Anyone may put into a phial a little coloured tincture, label it, etc., and call it a balm, tacking to it some nonsensical name, and an enormous price. Yet John Bull, who looks with such an economical scrutiny into his personal expenditure, will squander his wealth to such robbing humbuggers!" Or, again, "Where is the Vice Society that can incarcerate men in dungeons and fine them beyond their ability ever to pay for freely discussing theological questions, and yet, shame, shame, this vile race of plunderers! bloodsuckers! murderers! o'erspread and disfigure the land with impunity. And the public Press, too, where are their warnings? Alas! their patriotism is bartered, their love of mankind exchanged for their love of gold, and the wives and daughters of Englishmen are disgusted in the perusal of a newspaper by the filthy and abominable compositions of the quacks."

The extent to which the newspapers profited from announcements of the kind referred to in the last quotation was apparently very large even at that time, and, as may be imagined, their share in the traffic is occasionally referred to in the course of the campaign. Thus, it is stated that the "public journals are filled with the extraordinary successes of these men, their nostrums and their lies, and some of them have paid even as much as £4,000 a year for advertisements alone." The confidence which the *Medical Adviser* appears to have had in Parliament hardly seems to have been justified by facts. "We have laws passed against the adulteration of tea and coffee, and why not against such horrible compositions as the medicines of these unprincipled fellows, compounded of drugs of which they know nothing and of which the ablest physicians speak with doubt and caution?" That is a question still to be answered.

